# Botulinum toxin A modifies nociceptive withdrawal reflex in subacute stroke patients

**DOI:** 10.1002/brb3.1069

**Published:** 2018-08-23

**Authors:** Elena Alvisi, Mariano Serrao, Carmela Conte, Enrico Alfonsi, Cristina Tassorelli, Paolo Prunetti, Silvano Cristina, Armando Perrotta, Francesco Pierelli, Giorgio Sandrini

**Affiliations:** ^1^ Department of Neurophysiopathology IRCCS Casimiro Mondino National Neurological Institute Pavia Italy; ^2^ Department of Brain and Behavioural Sciences University of Pavia Pavia Italy; ^3^ Department of Brain Injury and Parkinson's Disease Rehabilitation Moriggia Pelascini Hospital Gravedona (Como) Italy; ^4^ Department of Medical and Surgical Sciences and Biotechnologies Sapienza Polo Pontino University of Rome Latina Italy; ^5^ Movement Analysis LAB Rehabilitation Centre Policlinico Italia Rome Italy; ^6^ Fondazione Don Gnocchi Milan Italy; ^7^ Department of Neurorehabilitation Casimiro Mondino National Neurological Institute Pavia Italy; ^8^ IRCCS Neuromed Pozzilli, Isernia Italy

**Keywords:** botulinum toxin A, nociceptive withdrawal reflex, spasticity, stroke, upper limb

## Abstract

**Objectives:**

The aims of this study were to evaluate the pattern of the nociceptive withdrawal reflex (NWR) of the upper limb at rest and after injection of Botulinum toxin type A (BoNT‐A) in poststroke subacute hemiparetic patients.

**Methods:**

Fourteen patients with poststroke subacute hemiparesis underwent clinical and instrumental evaluation and BoNT‐A injection. Painful electrical stimulation was applied to induce the NWR. Baseline EMG activity and NWR recordings (EMG and kinematic response) were performed at T0, one month (T1), and three months (T2) after the BoNT‐A injection, as were Modified Ashworth Scale (MAS) and Functional Independence Measure (FIM) scores.

**Results:**

Comparison of results at T0, T1, and T2 revealed significant changes in the MAS score for the elbow (*p* < 0.001) and wrist joints (*p* < 0.001) and in the FIM score at T0 and T2. BoNT‐A injection had a significant effect on both NWR amplitude and baseline EMG activity in the posterior deltoid (PD) and flexor carpi radialis (FCR) muscles as well as in all averaged muscles. Analysis of elbow kinematics before and after treatment revealed that the reflex probability rates were significantly higher at T1 and T2 than at T0.

**Conclusion:**

Injection of BoNT‐A in the subacute phase of stroke can modify both the baseline EMG activity and the NWR‐related EMG responses in the upper limb muscles irrespective of the site of injection; furthermore, the reflex‐mediated defensive mechanical responses, that is, shoulder extension and abduction and elbow flexion, increased after treatment. BoNT‐A injection may be a useful treatment in poststroke spasticity with a potential indirect effect on spinal neurons.

## 1 INTRODUCTION

Upper limb spasticity is a frequent complication of stroke that greatly interferes with hand function, motor relearning, recovery of voluntary movements, positioning, and hygiene; it can also cause pain, with activity limitation and loss of dexterity (Rosales et al., 2016; Sommerfeld, Eek, Svensson, Holmqvist, & von Arbin, 2003). For this reason, the pharmacological and rehabilitation treatment of spasticity is a primary objective in stroke patients (Bethoux, 2015). Botulinum toxin is routinely used to treat focal spastic hypertonia in various upper motor neuron disorders (Esquenazi et al., 2013; Simpson et al., 1996). Indeed, peripheral injection of botulinum toxin type A (BoNT‐A) is one available treatment for focal problematic spastic hypertonia and associated pain, and it is particularly effective on upper limb spasticity in the subacute phase after stroke (Gracies et al., 2015; Slawek, Bogucki, & Reclawowicz, 2005). Physiotherapy in the subacute phase can further prolong the positive effect induced by botulinum toxin injection (Han, Wang, Meng, & Qi, 2013; Kinnear, Lannin, Cusick, Harvey, & Rawicki, 2014). BoNT‐A acts at the neuromuscular junction, reducing the release of acetylcholine and thus decreasing muscle contraction. Although the primary effect of BoNT‐A occurs at the neuromuscular junction, resulting in chemodenervation, the toxin may also modify the sensory feedback loop, possibly acting at spinal cord level to induce central antinociceptive activity (Drinovac, Bach‐Rojecky, Matak, & Lacković, 2013). In addition to reducing spasticity, BoNT‐A may, by inhibiting the release of pain modulators (e.g. substance P), result in a decrease in pain (Arezzo, 2002).

Various experimental evidence, in both animals and humans, shows that both supraspinal structures and spinal circuitries undergo a drastic plastic rearrangement in the acute/subacute phases of stroke (Kerzoncuf et al., 2015). These plastic changes lead, on the one hand, to the development of spasticity, and, on the other hand, to motor recovery (Pin‐Barre & Laurin, 2015). In theory, treating spasticity with BoNT‐A from the early poststroke phase may improve the outcome of a rehabilitation intervention and further promote motor recovery (Slawek et al., 2005). From this perspective, it is extremely important to quantitatively evaluate spinal plastic changes induced by early BoNT‐A treatment. Electrophysiological measures, for example, the H‐reflex, have shown that upper limb segmental excitability shows marked changes across the different stages of stroke (Girlanda et al., 1997). Among the spinal reflexes, the nociceptive withdrawal reflex (NWR), which can be evoked by stimulating cutaneous sensory nerves, is known to be a sensitive tool for investigating sensory‐motor integration as well as nociception in both humans and animals (Sandrini et al., 2005). As NWR‐related EMG responses can be recorded from several muscles simultaneously, the NWR has the advantage of being a tool that can be used to explore spinal cord excitability at several metameric levels, for example, from C5 to C8 (Serrao et al., 2006, 2012). A previous study in chronic stroke patients with hemiparesis showed increased NWR‐related EMG and kinematic responses and impairment of upper limb NWR modulation (Bohannon & Smith, 1987; Serrao et al., 2012). It would be interesting to evaluate whether injection of BoNT‐A in the early phase of stroke modifies/modulates the excitability of NWR‐mediating neurons and involving several metameric levels irrespective to the injected muscles. Our assumption was that botulinum toxin, reducing spasticity at early stage, in patients who show some degree of muscle spasticity and residual motor activity, interferes with the plastic changes in the spinal cord neurons after stroke, and thus be useful for promoting a better motor recovery.

The aims of the present study were as follows: (a) to characterize the NWR pattern of the upper limb at rest in a sample of poststroke hemiparetic patients evaluated in the subacute phase; and (b) to evaluate the effects of BoNT‐A on NWR responses and EMG baseline activity.

## 2 MATERIALS AND METHODS

### 2.1 Patients

We enrolled patients with poststroke hemiparesis who met the following inclusion criteria: (a) an ischemic lesion of either the dominant or the nondominant hemisphere of the brain resulting in contralateral hemiparesis; (b) subacute phase of stroke (within 2–9 weeks of the ischemic event); (c) residual motor function in the upper limb muscles (Medical Research Council scale score: 2–3); (d) age 40–80 years; and (e) upper limb spasticity (Modified Ashworth Scale score ≥ 2 in at least two of the following: flexors of the elbow, wrist and fingers). The exclusion criteria were: (a) previous stroke; (b) cerebrovascular encephalopathy; (c) concurrent sensory deficits; (d) neglect; (e) apraxia; (f) cognitive deficit (Mini‐Mental State Examination score < 24); (g) muscle or joint pain; (h) concomitant orthopedic disorders of the affected arm; (i) aphasia.

All patients were evaluated by two experienced neurologists (GS, EA).

Muscle tone was evaluated using the 6‐point (0, 1, 1+, 2, 3, 4) Modified Ashworth Scale (MAS) (Bohannon & Smith, 1987), which has been shown to be useful for detecting muscle tone also in stroke patients. Its six points each express a different level of resistance of a relaxed limb to passive stretching (0: normal – 4: complete resistance with no movement). The Functional Independence Measure (FIM) (Keith, Granger, Hamilton, & Sherwin, 1987) was used as the outcome measure for independence in activities of daily living, considering both the total score and an upper limb subscore. The total FIM score ranges from 18 to 126, while the self‐care subscale, which is related to upper limb function and refers to activities such as feeding, putting oneself in order, washing and dressing, ranges from 5 to 35.

Of 24 patients screened, 14 (6 women, 8 men; mean age: 69.0 ± 7.0 years; age range: 53–79 years) met the inclusion criteria and were included in the study. The patients’ clinical characteristics are reported in Table 1.

**Table 1:** The patients’ clinical characteristics

Patient no.	Age (years)	Gender	Time since stroke (weeks)	Side of lesion	Site of ischemia
1	71	M	7	Right	Putamen, internal capsule
2	69	M	7	Left	Frontotemporal‐insular cortex
3	74	M	9	Right	Internal capsule
4	67	F	9	Left	Frontal cortex
5	56	F	9	Right	Caudate nucleus
6	71	M	6	Right	Frontotemporal cortex
7	75	M	7	Left	Temporoparietal‐occipital cortex, right
8	73	F	6	Left	Frontotemporal cortex, internal capsule
9	66	F	7	Left	Thalamus
10	79	M	9	Right	Frontotemporal‐parietal cortex
11	53	F	7	Right	Corona radiata, lateral ventricle
12	73	M	7	Left	Thalamus
13	71	F	7	Right	Frontotemporal‐parietal cortex
14	68	M	7	Left	Internal capsule

All patients were hospitalized in the Neurorehabilitation Unit of the Department of Neurology, C. Mondino National Neurological Institute, Pavia, Italy. During their hospital stay, the patients underwent a daily physiotherapy program conducted according to European stroke rehabilitation guidelines (Gillen, 2015).

The research was approved by the local ethics committee and complied with the Helsinki Declaration. All the subjects gave their written informed consent to participate.

### 2.2 BoNT‐A injection

The muscles to be treated were chosen on the basis of: the long experience of the injecting neurophysiologist (EA), data from the literature (RCP, 2009) and the tolerability profile of the BoNT‐A injected. Patients were injected with AbobotulinumtoxinA in doses ranging from a minimum of 50 UI to a maximum of 400 UI per site, for a total dosage per patient of between 450 and 1,680 UI. Table 2 shows the doses and injection sites for each patient treated with BoNT‐A. The injection was performed when the EMG pattern was characterized by involuntary tonic activity lasting longer than 500 ms (Fahn, 1987; Hallett, 1998).

**Table 2:** Doses and injection sites of BoNT‐A (Abobotulinum toxin A, UI)

Pt	BB	BR	PM	PT	TB	Br	FCU	FDP	FDS	FPL	ALP	FCR	TM	Total dosage
1	200	150	150	150			100	150	150	100				1,150
2	200	100		150		100	200	150	150					1,050
3	300	200	300	300				200	180	200				1,680
4				50				150	150	50			100	500
5	300		300	300								200		1,100
6				200				100	100	50				450
7				200			150		150			150		650
8	200			200					200		160	200		960
9	200	100	100	200			150	100	150					1,000
10			300	300			200							800
11				350	350				300	200				1,200
12	150		200	300		150			200					1,000
13	300	200	400	300										1,200
14	300		300						300					900

Note

ALP, abductor longus pollicis; BB, biceps brachii; Br, brachialis; BR, brachioradialis; FCR, flexor carpi radialis; FCU, flexor carpi ulnaris; FDP, flexor digitorum profundus; FDS, flexor digitorum superficialis; FPL, flexor digitorum superficialis pollicis longus; PM, pectoralis major; Pt, patient number; PT, pronator teres; TB, triceps brachii; TM, teres major.

### 2.3 Instrumental evaluation

Voluntary movement of the shoulder and the elbow joints was recorded using a 6‐camera optoelectronic system (ELITE, BTS Engineering, Milan, Italy) with a sampling rate of 100 Hz.

Ten markers were placed on anatomical landmarks in accordance with a validated upper limb and trunk biomechanical model (Rab, Petuskey, & Bagley, 2002). In detail, markers were placed over the left and right acromions, on the cutaneous projection of the spinous process of the seventh cervical vertebra, over the olecranon and the styloid ulnar and radial process on the paretic side, over the head of the third metacarpal bone, over the right and left anterior superior iliac spines, and over the sacrum.

Synchronized acquisition and data processing were performed using Capture and Analyzer software, respectively (BTS, Milan, Italy).

The surface EMG (sEMG) signals were recorded with a sampling rate of 1,000 Hz using a 16‐bit acquisition board, and amplified using a 16‐channel Wi‐Fi transmission surface electromyograph (Pocket EMG System, BTS, Milan, Italy). The common mode rejection ratio was 100 dB. After skin preparation, bipolar circular Ag/AgCl surface electrodes (FIAB SpA, Florence, Italy), prepared with electroconductive gel (diameter 1 cm, distance between the electrodes 2 cm), were placed in single differential configuration over the long head of the anterior and posterior deltoid (AD and PD), biceps brachii (BB), triceps brachii (TB), flexor carpi radialis (FCR), and extensor carpi radialis (ECR), according to standard anatomical landmarks.

Electrodes were placed on the center of each muscle belly, in the direction of the muscle fibers, according to European recommendations on sEMG (Hermens, Freriks, Disselhorst‐Klug, & Rau, 2000).

### 2.4 Painful electrical stimulation technique

We used the same electrical stimulation technique reported elsewhere (Don et al., 2008; Serrao et al., 2006). In brief, using ring electrodes, we stimulated the digital branch of the median nerve supplying the index finger. The stimulus was delivered by means of a constant‐current stimulator (Grass S‐88; Grass Medical Instruments, USA). It was composed of five pulses, each of 1 ms duration, released in a single stimulus train at 200 Hz. The pain threshold (PT) was identified using a staircase method (Willer & Bathien, 1977). The electrical stimulus intensity was then set at twice the PT (2xPT).

### 2.5 Procedure

Before the formal measurements began, the participants underwent a practice session to familiarize them with the experimental procedure.

For the NWR recordings, patients sat on a comfortable soft‐seated chair in a quiet, well‐lit room, in front of a table (height, 80 cm; width, 100 cm; depth, 60 cm). The subjects rested their arm on the table in a semiflexed position to encourage maximum muscle relaxation. In this position, the shoulder was abducted to 50° and anteflexed to 25°, the elbow was flexed to 100° with the forearm fully pronated, the wrist was in line with the forearm, and the fingers were spontaneously flexed. Electric shocks with an intensity of 2xPT were delivered to the patients in this position. Ten trials were recorded in each subject.

All patients were evaluated before (T0) and after one month (T1) and three months (T2) BoNT‐A injection. All BoNT‐A injections were performed by an experienced neurologist after careful evaluation of the muscles to be treated.

Nociceptive withdrawal reflex recordings (EMG and kinematic response) were performed at T0, T1 and T2. The MAS was administered at T0, T1 and T2, while the FIM was administered at T0 and T2.

### 2.6 Data analysis

The data were processed using 3D data processing software (SMART Analyzer, BTS, Milan, Italy). A validated biomechanical model was used to measure shoulder and elbow joint angles (Rab et al., 2002).

We analyzed the flexion and extension and the abduction and adduction of the shoulder joint as well as the flexion and extension of the elbow joint.

In order to evaluate the mechanical effect of the nociceptive stimulus, we analyzed the kinematic parameters in the 125–300 ms interval after the stimulation.

The sEMG signals were digitally filtered (bandwidth 20–400 Hz, 12 dB/oct roll‐off) in order to reduce slow transient noise and instrumentation noise. The sEMG signals recorded were processed using the following procedure: digital filtering (low pass Hamming filter with a cutoff frequency of 100 Hz), followed by rectification and smoothing using a root mean square with a mobile window of 10 ms.

We calculated the area under the curve both for the 140 ms interval of time immediately before the stimulus (baseline EMG activity/prestimulus interval) and for the 60–200 ms interval after the stimulus (poststimulus interval). The mean of the EMG area plus 2 *SD*, calculated in the prestimulus interval, was used as a threshold to determine the presence (denoted by a value above the threshold) or absence (a value below the threshold) of the NWR in the poststimulus interval in each trial. The NWR‐related EMG responses were measured as the difference between the EMG areas in the post‐ versus the prestimulus intervals for each examined muscle (Don et al., 2008; Serrao et al., 2006).

### 2.7 Statistical analysis

All the analyses were performed using SPSS statistics software (version 17).

One‐way ANOVA for repeated measures was performed to evaluate the effect of BoNT‐A injections on: baseline EMG activity and NWR‐related responses (EMG activity and kinematic reflex amplitudes), and Modified Ashworth Scale scores (three‐level within‐subject factors). We used Greenhouse–Geisser correction when appropriate to avoid violations of sphericity (Greenhouse & Geisser, 1959). The Bonferroni correction was used to counteract the problem of multiple comparisons.

The paired *t* test was used to compare the FIM scores between T0 and T2.

We calculated the reflex probability rate expressed as the percentage of trials in which the reflex‐related kinematic responses were present in a given joint (Table 3). The chi‐square test was used to compare the reflex probability rates in the different types of joint kinematic responses (shoulder flexion vs extension, shoulder abduction vs adduction and elbow flexion vs extension) in all subjects (Table 3).

**Table 3:** Kinematic analysis of reflex responses of the upper limb after painful stimulation of the index finger at baseline (T0), and 1 month (T1), and 2 months (T2) after treatment with botulinum toxin type A

Time	Shoulder joint			Elbow joint		
	Sagittal plane		Frontal plane		Sagittal plane	
	Flex.	Ext.	Abd.	Add.	Flex.	Ext.
T0	0%	**80.70%**	**74.10%**	0%	**81.30%**	39.40%
	———	2.6 ± 1.6°	2.8 ± 1.6°^a^	———	6.0 ± 2.4°^a^	4.8 ± 4.5°
T1	0%	**94.10%**	**94.40%**	0%	**95.40%**	25.00%
	———	3.0 ± 1.1°	3.2 ± 1.2°	———	7.5 ± 3.4°	6.0 ± 0.1**°**
T2	0%	**86.7.0%**	**84.40%**	0%	**87.2.%**	0%
	———	3.1 ± 1.2°	4.2 ± 2.0°^a^	———	9.9 ± 3.7**°** a	———

Notes

Data are expressed as percentage values of 140 trials and as range of motion (°) expressed as mean ± *SD*.

a Indicates significant differences at post hoc analysis (*p* < 0.05).

The Spearman correlation test was used to correlate the reflex‐related EMG responses with the total dosage of BoNT‐A.

We used a significance level of *p* < 0.05.

## 3 RESULTS

### 3.1 Scale scores

A significant effect of BoNT‐A injections on MAS scores was found for the elbow (main effect, *F*_(2,26)_ = 71.401, *p* < 0.001) and wrist joints (main effect, *F*_(2,26)_ = 23.735, *p* < 0.001), while no significant differences were found for the shoulder joint. Post hoc analysis revealed lower levels of spasticity at both T1 (1.1 ± 0.2) (*p* < 0.001) and T2 (1.1 ± 0.4) compared to T0 (2.0 ± 0.4) (*p* < 0.001) for the elbow joint; lower values at both T1 (1.2 ± 0.3 *p* < 0.001) and T2 (1.8 ± 0.5 *p* < 0.001) as compared to T0 (2.2 ± 0.8) was also found for the wrist joint. Furthermore, at the wrist joint we recorded a significant increase in spasticity at T2 (1.8 ± 0.5) as compared to T1 (1.2 ± 0.3) (*p* < 0.05).

Significant differences in both FIM total score and FIM upper limb subscore were found between T0 (total FIM: 58.5 ± 26.4; upper limb subscore: 5 ± 0) and T2 (total FIM: 78.5 ± 23; upper limb subscore: 11.7 ± 4.5) (all, *p* < 0.001).

### 3.2 Baseline EMG activity and EMG reflex responses

**Figure 1:**
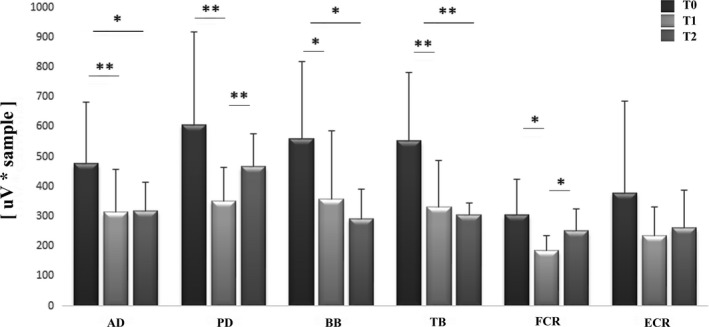
Baseline EMG activity in the AD, PD, BB, TB, FCR, and ECR muscles in a representative subject at baseline (T0) and 1 month (T1) and 3 months (T2) BoNT‐A injection. *p < 0.05 and **p < 0.01 at post hoc analysis

A significant effect of BoNT‐A injections on the baseline EMG activity was found in all the muscles but the ECR (Table 4). Post hoc analysis revealed significantly lower values at T1 and/or T2 compared to T0 in all muscles and significant lower values at T1 compared to T2 in PD and FCR muscles (Figure 1).

**Table 4:** Baseline EMG activity before (T0) and after (T1 and T2) BoNT‐A injection

Muscles	T0		T1		T2		ANOVA for repeated measures			
	Mean (uV•sample)	*SD*	Mean (uV•sample)	*SD*	Mean (uV•sample)	*SD*	*df*		*F*	*p*
AD	476.2	207.4	314.2	143.1	319	96	1.184	15.388	7.58	**0.012**
PD	607.2	310.7	351.3	113.7	465.8	111.7	1.129	14.672	9.85	**0.006**
BB	558.6	260.2	357.5	227.5	292.5	99.6	2	26	6.43	**0.005**
TB	552.2	230.4	332	155	304.2	38.5	2	26	15.09	**0.001**
FCR	304	120.9	184.2	50	250.8	74.6	2	26	6.16	**0.006**
ECR	377.8	306.2	235	96.1	260.5	128.4	1.367	17.766	2.21	0.15

**Figure 2:**
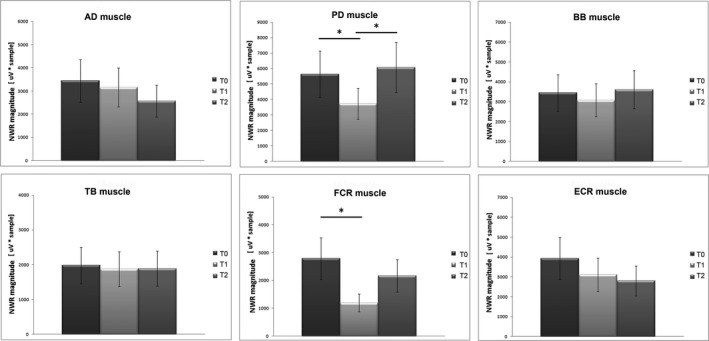
Nociceptive withdrawal reflex (NWR) amplitudes of AD, PD, BB, TB, FCR, and ECR muscles in all patients. The figure shows the mean and standard deviation values of the NWR amplitude before (T0) and 1 month (T1) and 3 months (T2) after BoNT‐A injection. *p < 0.05, at post hoc analysis

A significant effect of BoNT‐A injections was found on the NWR amplitude in the PD muscle (*F*_(2,26)_ = 5.627, *p* = 0.009). Post hoc analysis revealed lower values at T1 versus both T0 and T2, whereas no differences were found between T0 and T2 (Figure 2).

A significant effect of BoNT‐A injections was found on the NWR‐related EMG responses amplitude in the FCR muscle (*F*_(1.352, 17.576)_ = 8.849, *p* = 0.005). Post hoc analysis revealed lower values at T1 compared to T0, whereas no significant differences were found between T0 and T2 or T1 and T2 (Figure 2).

No significant effect of BoNT‐A injections was found on NWR‐related EMG amplitude in the AD, BB, TB and ECR muscles (*p* > 0.05), although a trend toward NWR‐related EMG amplitude reduction was evident for all these muscles (Figure 2).

**Figure 3:**
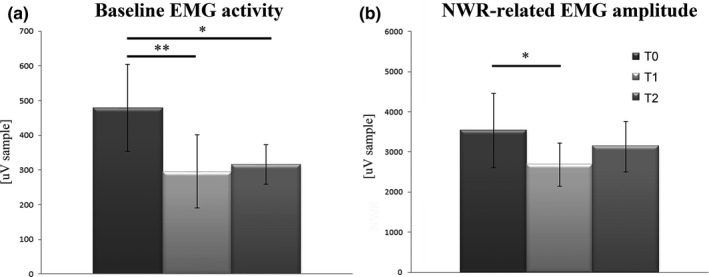
Mean baseline EMG activity in all patients before (T0) and 1 month (T1) and 3 months (T2) after BoNT‐A injection (a); mean nociceptive withdrawal reflex‐related EMG amplitude of all muscles in all patients before (T0) and 1 month (T1) and 3 months (T2) after BoNT‐A injection (b). *p < 0.05, at post hoc analysis

BoNT‐A injections showed a significant effect on the baseline EMG activity when averaged across all muscles (*F*_(2,26)_ = 22.643, *p* = 0.001). Post hoc analysis revealed significantly lower values at T1 and T2 compared with T0 (Figure 3a).

BoNT‐A injections showed a significant effect on the reflex‐related EMG responses when these were averaged across all muscles (*F*_(2,26)_=4.934, *p* = 0.015). Post hoc analysis revealed significantly lower values at T1 compared with T0 (Figure 3b).

A significant correlation was found between the averaged NWR‐related EMG responses and the total dosage of BoNT‐A (*r* = −0.736, *p* = 0.01).

### 3.3 Reflex‐related kinematic responses

Analysis of elbow kinematics before and after treatment revealed that the reflex probability rates (Table 3) were significantly higher both at T1 and T2 than at T0 (chi‐square test, for all, *p* < 0.05). Although a trend toward recovery of the baseline conditions at T2, no significant differences were found between T1 and T2 (Table 3).

The most common reflex kinematic pattern, at all three time points, consisted of shoulder extension (probability rate >80%), shoulder abduction (probability rate > 74%) and elbow flexion (probability rate > 81%) (Table 3).

A significant effect of BoNT‐A injections was found for shoulder abduction (ANOVA main effect, *F*_(2,26)_ = 5.760, *p* = 0.008) and elbow flexion (ANOVA main effect, *F*_(2,26)_ = 7.841, *p* = 0.002), whereas no effect was found for shoulder extension (main effect, *F*_(1.322, 17.185)_ = 1.418, *p* = 0.260). Post hoc analysis revealed significantly higher shoulder abduction and elbow flexion angle values at T2 compared to T0 (Table 3).

**Figure 4:**
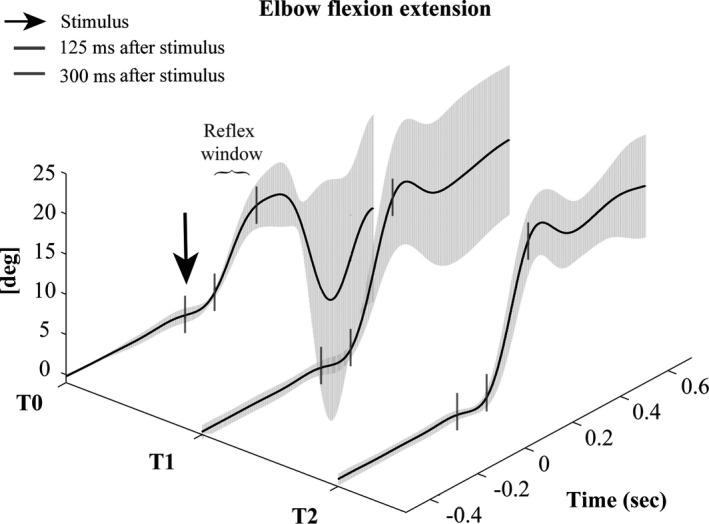
Kinematic reflex responses of the elbow (flexion–extension) in a representative subject at baseline (T0) and 1 month (T1) and 3 months (T2) after BoNT‐A injection. The black arrow indicates the stimulus delivery, and the gray dashes indicate the reflex detection windows. Note the increased elbow flexion at T1 and T2

**Figure 5:**
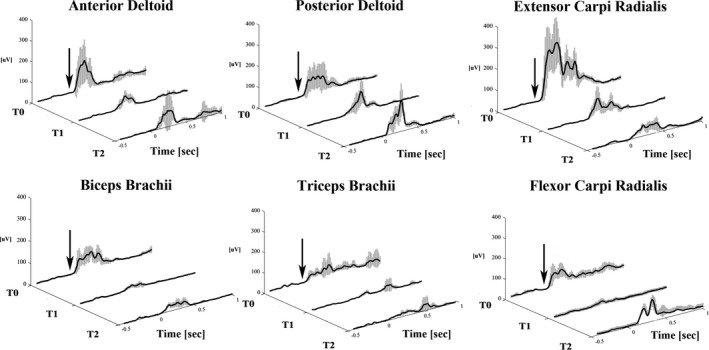
EMG reflex responses of the PD, BB, TB, FCR, and ECR muscles in a representative subject before (T0) and 1 month (T1) and 3 months (T2) after BoNT‐A injection. The black lines represent the mean curves, while the gray bands represent the standard deviation. The black arrows indicate the stimulus delivery

The changes in the NWR‐related responses (kinematic and EMG) after BoNT‐A injection in a representative patient are reported in Figures 4 and 5.

## 4 DISCUSSION

In the current study, the upper limb NWR was used as a tool to evaluate the effect of BoNT‐A administration on the excitability of spinal cord neurons in the subacute phase of stroke. In particular, our aim was first to characterize abnormal patterns of the multijoint, multimuscle NWR, and then to evaluate the changes in this reflex induced by injection of BoNT‐A. Our assumption was that this investigation may help to shed light both on the plastic rearrangement occurring at the level of the NWR‐mediating neurons, and on the changes induced by BoNT‐A injection that may, potentially, prevent the development of spasticity and promote a better motor recovery.

The main results can be summarized as follows: (a) the spasticity, as measured by MAS scores, was significantly reduced at both elbow and wrist joints level after BoNT‐A injection at one (T1) and three (T2) months, with a trend toward recovery of the baseline condition at three months (T2) in the wrist joint; (a) baseline EMG activity was significantly reduced in all muscles, when considered as both single and averaged values, at one (T1) and/or three (T2) months after BoNT‐A injection with a trend toward recovery of the baseline condition at three months (T2) only for PD and FCR muscles; (b) reflex‐related EMG responses were significantly reduced in the PD and FCR muscles, as well as in all the muscles (when their values were averaged) at one month (T1) after BoNT‐A injection, after which there was a trend toward recovery of the baseline condition at three months (T2); (c) oppositely to reflex‐related EMG responses, the shoulder and elbow reflex‐related kinematic responses were significantly increased at both one (T1) and three (T2) months after BoNT‐A injection, with a trend toward recovery of the baseline condition at three months (T2).

Only a few studies have investigated changes in NWR excitability in poststroke hemiparetic patients with spasticity in the lower (Rueterbories, Spaich, & Andersen, 2013; Spaich, Hinge, Arendt‐Nielsen, & Andersen, 2006; Spaich, Svaneborg, Jørgensen, & Andersen, 2014) or upper (Serrao et al., 2012) limbs. These previous studies focused on the chronic phase of stroke, whereas to date no studies have evaluated patients in the acute/subacute phase. We found that patients, within 2–9 weeks of stroke, showed a mechanical reflex response characterized by shoulder extension and abduction associated with elbow flexion. This abnormal mechanical response remained unchanged at subsequent evaluations. The reflex pattern we found is similar to that reported by Dewald and colleagues (Dewald, Beer, Given, McGuire, & Rymer, 1999), although they found shoulder adduction instead of abduction, and it is in line with the classical description of poststroke evolution (Brunnström, 1970), characterized by the presence of abnormal motor synergies.

With regard to the NWR‐related EMG responses after treatment, a significant decrease in PD and FCR muscles, as well as in the averaged responses of all muscles, were found at 1 month (T1). A similar decrease was observed also for the baseline EMG activity. Both NWR‐related EMG amplitude and baseline EMG activity changes were likely induced by BoNT‐A and reflect the fact that their maximal clinical effect occurs about one month after the injection (Gordon et al., 2004). The reduction of the spasticity at the elbow and wrist joints one month after injection, as revealed by reduction of the MAS scores, further confirms the causal effect of BoNT‐A in reducing both the NWR‐related EMG responses and baseline EMG activity. Such suppressive action is directly exerted by the BoNT‐A on the muscle by blocking the nicotine receptors (Poulain, Lonchamp, Jover, Popoff, & Molgó, 2009). However, as the BoNT‐A injection reduced the EMG amplitudes also in noninjected muscles (e.g. PD), we cannot completely exclude a global inhibitory effect of BoNT‐A on the cervical spinal neurons. Such inhibition may be due to a retrograde action of the toxin, which could modify the sensory feedback loop and act at spinal cord level as shown by previous studies (Drinovac et al., 2013). In addition, BoNT‐A by blocking <alpha> and <gamma> fibers could modify several sensory afferents and indirectly change the baseline EMG activity and the NWR EMG responses possibly due to plastic changes at spinal level. The fact that the inhibitory effect was found mainly in PD and FCR muscles may be explained by the important role these muscles play in the withdrawal reflex as well as in the pathologic flexor synergy development. In this view, the BoNT‐A may reduce the excitability of the cervical spinal neurons involved in both the flexor reflex and pathologic flexor synergy, by mainly attenuating their strongest components (e.g. PD and FCR muscles). All of a sudden, although a trend for a reduction at 1 month, the BB muscle, which should also have an important role in both the withdrawal reflex and flexor synergy development at the elbow joint, was not significantly reduced. This could be explained by the fact that several other, not investigated, muscles (i.e. brachioradialis, brachial, and coracobrachialis muscles) may concur in such a role. The lack of the inhibitory effect of BoNT‐A on the NWR‐related EMG responses at 3 months (T2) (Figures 1 and 2), together with the re‐increase of the spasticity at wrist joint level, strongly suggests a loss of the effect of the BoNT‐A at 3 months (Kinnear et al., 2014). In this regard, the NWR may be a useful adjunctive tool to follow the temporal evolution of the BoNT‐A injection effect.

On the contrary, the NWR‐related kinematic responses were not reduced at 1 month (T1) after BoNT‐A injection, but instead tended to increase in terms of both reflex probability rate and amplitude (Table 3). This increase may be due to the spontaneous changes occurring in the poststroke period, but also to the reduction in EMG activity (muscle relaxation), versus baseline, that was induced by BoNT‐A injection and observed at T1 and/or T2. The exaggerated mechanical responses in stroke patients may thus be the result of a complex interaction of upper limb antagonist and synergistic muscles. Indeed, BoNT‐A injection may help to reactivate reciprocal group Ia interneurons possibly by blocking Renshaw cells (Marchand‐Pauvert et al., 2013). Nevertheless, as, in stroke, the pathological flexor synergies are stronger than the extensor synergies (Brunnström, 1970), the increased reflex mechanical responses may be interpreted as the natural consequence of the muscle relaxation/inhibition. Whether higher doses, in more muscles, can either modify or reverse such mechanical responses needs to be addressed in further studies. An alternative interpretation of our findings is that

Most of the studies on botulinum toxin injection for poststroke spasticity have been performed in chronic stroke patients. However, early use of botulinum toxin (in acute‐to‐subacute stroke) has been shown to reduce spasticity, preventing disabling stiffness and contractures (Ada, O'Dwyer, Ada, O'Dwyer, & O'Neill, 2006; Chen, 2012; Hesse et al., 2012). A recent study showed that botulinum toxin injection was more helpful for spasticity, contractures, and function in subacute patients than in chronic ones (Lim, Choi, & Lim, 2016). Our study showed that injection of BoNT‐A in the subacute phase of stroke can reduce baseline EMG activity and the NWR‐related EMG reflex responses in the upper limb muscles which, in turn, suggests that BoNT‐A may change the abnormal EMG pattern characterizing the NWR. Taken together, these findings suggest introducing BoNT‐A early may be more useful than delaying the treatment, and should therefore probably be encouraged. From this perspective, the NWR could prove to be a useful tool for evaluating the effect of the BoNT‐A in stroke patients.

Some of the limitations of this study include the lack of a comparison with physiotherapy alone and/or with a placebo group. Thus, randomized controlled studies are needed to confirm our findings. In addition, further studies are warranted to verify, in a longer follow‐up, whether combining repeated BoNT‐A injections (from the acute through to the chronic phase of stroke) with physiotherapy might modify the reflex responses and improve the motor recovery. Furthermore, how much the rehabilitation program may have affected the patient cohort remains speculative here. Hence, a nonintervention cohort (Rehab program only) vs BTX with or without Rehab will be an ideal approach.

## CONFLICT OF INTEREST

None of the authors have potential conflict of interests to be disclosed.
